# The effects of a composition of ginseng polysaccharides and *Houttuynia cordata* essential oil on the growth performance, slaughter performance, serum biochemistry and intestinal health of Arbor Acres broilers

**DOI:** 10.3389/fvets.2026.1864942

**Published:** 2026-06-26

**Authors:** Weiwei Wang, Zhaobin Zhou, Sijuan Huang, Denghui Hu, Bin Zhou, Huiran Kang, Jieyang Wu, Zikui Liu

**Affiliations:** 1 College of Veterinary Medicine, Hunan Agricultural University, Changsha, Hunan, China; 2 Hunan Microorganism & Herb Biotechnology Co., Ltd., Xiangtan, Hunan, China; 3 Academic Affairs Research Office, Shaoyang Industry Polytechnic College, Shaoyang, Hunan, China

**Keywords:** antibiotic, growth performance, *Houttuynia cordata* essential oil, intestinal microbiota, *Panax ginseng* polysaccharide

## Abstract

**Introduction:**

This study compared the efficacy of a *Panax ginseng* polysaccharide (GPS) and *Houttuynia cordata* essential oil (HCEO) complex with antibiotics in Arbor Acres broilers.

**Methods:**

Three hundred 1-day-old broilers were randomly assigned to five groups: a blank control group (C), an antibiotic group (A), and three groups administered the GPS-HCEO complex via drinking water at 0.1 (M1), 0.3 (M2), or 0.6 mL/L (M3).

**Results:**

Compared with Group C, FCR was significantly decreased in Groups M1, M2, and M3 during the early phase (*P* < 0.05). During the late phase and the overall experimental period, FCR was significantly lower in Groups A, M1, and M3 than in Group C (*P* < 0.05). For slaughter performance, the four treatment groups showed no significant differences in slaughter rate, semi-eviscerated rate, eviscerated rate, breast muscle rate, leg muscle rate, lean meat rate, or wing rate compared with Group C (*P* > 0.05), whereas Group A had a significantly lower abdominal fat rate than Group C and the three GPS-HCEO groups (*P* < 0.05). Serum analysis showed that IgG, IgM, IgA, IL-1, and IL-10 levels remained unchanged compared with Group C (*P* > 0.05), whereas serum triglyceride levels were increased in all treatment groups (*P* < 0.05). Antibiotic supplementation impaired duodenal morphology and reduced cecal microbial diversity, as reflected by the Simpson index, as well as the relative abundance of *Firmicutes* and *Tenericutes*. Supplementation with 0.6 mL/L GPS-HCEO complex achieved a feed conversion efficiency comparable to that of antibiotics. Unlike antibiotics, the GPS-HCEO complex improved jejunal and ileal morphology, as indicated by increased VH and V/C ratio, without disrupting the core microbial structure. It also promoted the enrichment of the beneficial genera *Aerococcus* and *Jeotgalicoccus* in the cecum.

**Conclusions:**

These findings suggest that the GPS-HCEO complex has potential as a feed additive for reducing antibiotic use in broiler production.

## Introduction

Globally, antibiotics are widely used in livestock and poultry farming, primarily for disease prevention, Treatment, and growth promotion, making significant contributions to the rapid development of the industry (1). However, the long-term abuse of antibiotics has caused serious public safety issues. On one hand, it enhances the drug resistance of animal-derived bacteria, increasing the difficulty of treating bacterial diseases (2); on the other hand, resistance genes threaten human health through residues in meat products and fecal discharge (3). As an increasing number of countries emphasize antibiotic substitution in animal feed and breeding, the development of safe and effective alternatives is particularly important for improving livestock growth performance and production efficiency.

*Panax ginseng* polysaccharide (GPS) is an important prebiotic that can promote the proliferation of beneficial bacteria to improve intestinal health. Additionally, it regulates immune function by modulating the activity of various immune cells (4). GPS has been widely used in livestock production; for instance, Liu (5) found that adding 200 g/T GPS to the diet improved the growth performance and immune indices of Xuefeng black-bone chickens and reduced diarrhea and mortality rates. Similarly, Yang (6) showed that in pigs, the addition of 800 g/T GPS enhanced growth performance by improving intestinal morphology and increasing intestinal short-chain fatty acid content. However, the direct antibacterial activity of GPS is relatively limited, whereas essential oil-derived substances have been widely recognized internationally for their direct antibacterial effects. Houttuynia cordata essential oil (HCEO) is the volatile oil derived from Houttuynia cordata, with sodium houttuyfonate as its core component, which exhibits positive antibacterial and anti-inflammatory effects (7). Studies (8) have shown that adding 1,000 g/T Houttuynia cordata extract to feed helps improve growth performance and apparent nutrient digestibility in goats, enhancing antioxidant, immune, and anti-inflammatory responses. Moreover, a compound preparation composed of Houttuynia cordata, Curcuma longa, and other components has been reported to improve the survival rate of broilers infected with Salmonella (9). Yan (10) also found that adding 1,000 g/T Houttuynia cordata extract significantly reduced the abundance of *E. coli* in the pig intestine without affecting the abundance of *Lactobacillus*.

The effect of replacing antibiotics with a single active ingredient is often limited. Based on the principle of mechanistic complementarity, the synergistic application of multiple active ingredients offers broader prospects for antibiotic substitution (11). GPS and HCEO possess excellent complementarity in their mechanisms of action; therefore, we hypothesized that their synergistic combination could serve as an effective antibiotic alternative. Arbor Acres broilers are fast-growing birds promoted globally, and antibiotics are widely used in their breeding. Consequently, this study aimed to compare the application effects of a GPS and HCEO complex vs. antibiotics on Arbor Acres broilers, analyzing growth performance, slaughter performance, serum biochemistry, immune function, inflammatory factors, intestinal morphology, and microbiota composition.

## Materials and methods

### Ethics statement

The Animal Ethics Committee of Hunan Agricultural University (Changsha, China) reviewed and approved all experimental protocols (HAU ACC 2022176).

### Preparation of the GPS-HCEO complex and experimental diets

The GPS-HCEO compound was prepared by mixing GPS and HCEO at a weight ratio of 9:1, diluted with nine times the weight of distilled water, and then emulsified with 1% Tween-80 to obtain the GPS-HCEO complex. Multiple assays confirmed that the final product contained 8% GPS and 1.2% sodium houttuyfonate. The remaining formulation mainly consisted of distilled water, 1% Tween-80, and minor volatile constituents of HCEO. The basal det used in the experiment was provided by the Hunan Institute of Animal Husbandry. The antibiotic diet was prepared by adding 50 g/T chlortetracycline (The effective content is 20%) and 50 g/T salinomycin (The effective content is 20%) to the basal diet. The composition and nutrient levels of the basal diet are shown in Table 1.

**Table 1:** Composition and nutrient levels of basal diets (air-dry basis).

Items	%
Corn	63
Soybean meal	22
Wheat bran	0.3
Corn protein meal	7
Soybean oil	1.6
Limestone	1.4
Ca(HCO3)2	2
NaCL	0.2
Premix^1)^	2.5
Total	100
Nutrients	
CP	23
Ca	1
P	0.5
ME(MJ/kg)	13
Methionine	0.5
Lysine	1.5

^1)^The premix provided per kilogram of diet: vitamin A (retinyl acetate), 12,000 IU; vitamin D3 (cholecalciferol), 2,500 IU; vitamin E (DL-α-tocopheryl acetate), 20 IU; menadione, 1.3 mg; thiamin, 2.2 mg; riboflavin, 8.0 mg; nicotinamide, 40 mg; choline chloride, 400 mg; calcium pantothenate, 10 mg; pyridoxine HCl, 4 mg; biotin, 0.04 mg; folic acid, 1 mg; vitamin B12 (cobalamin), 0.013 mg; Fe (from ferrous sulfate), 80 mg; Cu (from copper sulfate), 8.0 mg; Mn (from manganese sulfate), 110 mg; Zn (from zinc sulfate), 60 mg; I (from calcium iodate), 1.1 mg; Se (from sodium selenite), 0.3 mg.

### Animal husbandry and experimental design

Three hundred healthy 1-day-old Arbor Acres (AA) broilers (38.66 ± 0.61 g/bird) were selected and randomly divided into five treatment groups, with three replicates per group and 20 birds per replicate. Each replicate included two cages: one cage containing 10 male broilers and one cage containing 10 female broilers. The five groups were: Blank Control (Group C), Antibiotic Group (Group A), and three experimental groups administered the GPS-HCEO complex via drinking water at Low (Group M1, 0.1 ml/L), Medium (Group M2, 0.3 ml/L), and High (Group M3, 0.6 ml/L) doses. Group C and the three M groups were fed the antibiotic-free basal diet, while Group A was fed the diet supplemented with chlortetracycline and salinomycin. The experiment lasted for 42 days, divided into the early phase (0–21 days) and the late phase (21–42 days). The birds were raised in cages with *ad libitum* access to feed and water. Feeding occurred twice daily. Temperature, humidity, vaccination program and lighting conditions were strictly maintained according to standard Arbor Acres broiler management procedures, with regular cleaning, disinfection, and ventilation of the chicken coop.

### Growth performance

Broilers were weighed at 1, 21, and 42 days of age (2 birds per replicate, half male and half female) to calculate the average body weight, recorded as BW0, BW21, and BW42, respectively. Average daily gain (ADG) was calculated as (BWd—BW0)/d. Feed intake was recorded over 21 days by weighing offered and residual feed. Average daily feed intake (ADFI) was calculated as (Total feed offered–Total residual feed)/21. The feed conversion ratio (FCR) was calculated based on ADG and ADFI.

### Slaughter performance

At 42 days of age, two birds with body weights close to the average body weight of each replicate were selected, including one male and one female bird. After fasting for 12 (with water available), blood was collected, and the birds were slaughtered. Carcass weight, semi-eviscerated weight, eviscerated weight, breast muscle weight, leg muscle weight, lean meat weight, wing weight, and abdominal fat weight were measured according to the “Terminology and Measurement Methods for Poultry Production Performance” (NY/T 823-2020). Corresponding percentages (slaughter rate, semi-eviscerated rate, eviscerated rate, breast muscle rate, leg muscle rate, lean meat rate, wing rate, and abdominal fat rate) were calculated.

### Serum biochemical indices

After the 42-day trial, blood was drawn from the wing veins of two selected birds per replicate. Serum was separated by centrifugation and stored at −80 °C. A Mindray BS-240VET automatic animal biochemical analyzer (kits purchased from Nanjing Jiancheng Bioengineering Institute) was used to measure alanine aminotransferase (ALT), aspartate aminotransferase (AST), total cholesterol (TC), creatinine (CREA), urea (UREA), glucose (Glu-G), triglycerides (TG), total protein (TP), albumin (ALB), and globulin (Glo).

### Immune indices and inflammatory factors

Serum collected at the end of the trial was analyzed for IgM, IgG, IgA, IL-1, and IL-10 levels using ELISA kits according to the manufacturer's instructions (Nanjing Jiancheng Bioengineering Institute, China).

### Small Intestine morphology

At 42 days, segments of the duodenum, jejunum, and ileum were collected, fixed, washed, cleared in xylene, embedded in paraffin, sectioned, and stained with hematoxylin-eosin (HE). Images of villi, crypts, and muscle layers were captured under an optical microscope. Villus height (VH) and crypt depth (CD) were measured using the Mshot Digital Microscopy Imaging System, and the villus height/crypt depth ratio (V/C) was calculated.

### Cecal microbiota composition

After the 42-day trial, cecal digesta samples were collected from two birds in each replicate, immediately frozen in liquid nitrogen, and stored at −80 °C until further analysis. Total genomic DNA was extracted from the cecal digesta samples using the PowerSoil^®^ DNA Isolation Kit (QIAGEN, Germany). DNA quality and concentration were assessed using a NanoDrop ND-2000 spectrophotometer (Thermo Fisher Scientific Co., Ltd., China). Qualified DNA samples were then submitted to Shanghai Personal Biotechnology Co., Ltd. for library construction and high-throughput sequencing. Paired-end sequencing of community DNA fragments was performed on an Illumina platform with a read configuration of 2 × 250 bp. The V3–V4 hypervariable region of the bacterial 16S rRNA gene was amplified using the primer pairs 338F (5′-ACTCCTACGGGAGGCAGCAG-3′) and 806R (5′-GGACTACHVGGGTWTCTAAT-3′) in a T100 Thermal Cycler PCR system (Bio-Rad, USA). PCR products were extracted from 2% agarose gels, purified using a PCR Clean-Up Kit (YuHua, Shanghai, China) according to the manufacturer's instructions, and quantified using a Qubit 4.0 fluorometer (Thermo Fisher Scientific Co., Ltd., China). Sequencing data were processed using standard bioinformatic procedures. Tags were clustered into operational taxonomic units (OTUs) at a 97% sequence similarity threshold using USEARCH software. Taxonomic annotation of OTUs was performed against the SILVA reference database. Bioinformatics analysis was performed for Alpha diversity and species composition. To identify the bacterial taxa that were differentially represented at the genus or higher taxonomic levels, linear discriminant analysis coupled with effect size (LEfSe) was performed, where linear discriminant analysis (LDA) method was used to rank the features differing between the groups, and a LDA score >3 was considered significant.

### Statistical analysis

Statistical analysis was performed with SPSS 22.0 software (IBM, USA). Data were tested for normal distribution using the Kolmogorov–Smirnov (K-S) test. The non-normally distributed data was transformed using their respective arctangent, while the normally distributed data was left unchanged. Differences between groups were examined using one-way analysis of variance (ANOVA) with Duncan's *post hoc* test (the model included treatment as the fixed effect, with treatment df = 4 and error df = 10), when the homogeneity of variance is not significant (*P* > 0.05). When the homogeneity of variance is significant (*P* < 0.05), using Welsh ANOVA test with Tamhane *post hoc* test. The linear and quadratic effects of dietary GPS-HCEO complex supplementation dose were evaluated by regression analysis. All the results were expressed as mean ± standard deviation (SD). Differences were considered significant at *P* < 0.05.

## Results

### Growth performance

As shown in Table 2, in the early phase, the FCR of Group A decreased by 0.05 compared to Group C, and Groups M1, M2, and M3 decreased by 0.12, 0.14, and 0.14, respectively, though these differences were not statistically significant (*P* > 0.05). In the late phase, the FCR of Group A and the three GPS-HCEO groups was significantly lower than that of Group C (*P* < 0.05), with the M groups showing FCRs close to Group A. Over the full period, adding antibiotics or the GPS-HCEO complex did not significantly affect ADFI or ADG compared to Group A, but there was a trend toward reduced FCR (0.05 < *P* < 0.1).

**Table 2:** Effect of GPS and HCEO complex on growth performance of broilers.

Item	Treatment group					***P-value***		
	C	A	M1	M2	M3	ANOVA	Linear	Quadratic
Pre-experiment								
BW0 (g)	38.58 ± 0.69	38.82 ± 0.57	38.97 ± 1.02	38.54 ± 0.55	38.56 ± 0.27	0.904	0.794	0.656
BW21 (g)	550.22 ± 19.07	561.39 ± 0.40	540.29 ± 23.10	562.22 ± 3.96	579.25 ± 23.69	0.146	0.059	0.259
ADFI (g/d/bird)	46.54 ± 3.14^a^	46.20 ± 0.35^a^	42.76 ± 0.05^b^	44.31 ± 0.15^ab^	44.37 ± 0.49^ab^	0.049	0.294	0.061
ADG (g/d/bird)	24.13 ± 0.95	24.55 ± 0.38	23.62 ± 1.07	24.68 ± 0.17	25.49 ± 1.08	0.148	0.057	0.240
FCR	1.96 ± 0.07^a^	1.91 ± 0.02^ab^	1.84 ± 0.09^b^	1.82 ± 0.03^b^	1.81 ± 0.05^b^	0.022	0.016	0.110
Post-experiment								
BW42 (g)	1,904.80 ± 27.80	2,020.81 ± 124.78	2,001.66 ± 142.48	1,953.53 ± 112.09	2,089.73 ± 173.48	0.488	0.159	0.794
ADFI (g/d/bird)	151.75 ± 5.49	139.19 ± 13.12	147.34 ± 6.35	148.79 ± 5.49	152.92 ± 11.87	0.425	0.811	0.369
ADG (g/d/bird)	64.50 ± 2.24	69.50 ± 5.93	69.59 ± 5.69	66.25 ± 5.15	71.93 ± 7.14	0.514	0.208	0.926
FCR	2.38 ± 0.01^a^	2.15 ± 0.10^bc^	2.13 ± 0.08^c^	2.26 ± 0.08^ab^	2.09 ± 0.05^c^	0.003	0.001	0.332
Full-experiment								
ADFI (g/d/bird)	99.14 ± 1.15	92.69 ± 6.78	95.05 ± 2.88	96.55 ± 2.16	98.64 ± 3.06	0.270	0.998	0.059
ADG (g/d/bird)	44.31 ± 0.65	46.70 ± 3.04	46.60 ± 3.37	45.47 ± 2.66	48.71 ± 4.11	0.503	0.157	0.790
FCR	2.24 ± 0.04^a^	2.09 ± 0.07^b^	2.06 ± 0.08^b^	2.14 ± 0.06^ab^	2.05 ± 0.09^b^	0.036	0.024	0.237

Data are presented as least squares means based on 3 replicate observations per treatment, with each observation representing the average value of the corresponding measurements from one male and one female bird within each replicate. BW, body weight; ADFI, average daily feed intake; ADG, average daily gain; FCR, feed conversion ratio; SEM, standard error of means. ^1)^C, a blank control (basal diets); A, an antibiotic group (basal diets +50 g/T chlortetracycline + 50 g/T salinomycin); M1, basal diets+ GPS and HCEO complex via drinking water at 0.1 ml/L; M2, basal diets+ GPS and HCEO complex via drinking water at 0.3 ml/L; M3, basal diets+ GPS and HCEO complex *via* drinking water at 0.6 ml/L. ^2)^The total *p*-value was obtained from the ANOVA analysis conducted on the 5 groups. The *p*-values for the linear and quadratic terms were derived from the ANOVA analysis performed on the control group and the 3 dose groups. ^a−*c*^Means within a variable with no common superscript differ significantly (*p* < 0.05).

As presented in Table 2, ADG was not significantly affected by dietary treatment throughout the experimental period compared with Group C (*P* > 0.05). In the early phase, Group M1 showed a lower ADFI than Group A (*P* < 0.05), whereas ADFI did not differ significantly between the four treatment groups and Group A during the late phase or the overall period (*P* > 0.05). Compared with Group C, FCR was significantly reduced in Groups M1, M2, and M3 during the early phase (*P* < 0.05). In the late phase and over the entire experimental period, Groups A, M1, and M3 exhibited significantly lower FCR than Group C (*P* < 0.05).

### Slaughter performance

Table 3 indicates that compared to Group C, neither Group A nor the Groups M1, M2, M3 showed significant differences in slaughter rate, semi-eviscerated rate, eviscerated rate, breast muscle rate, leg muscle rate, lean meat rate, or wing rate (*P* > 0.05). However, the leg muscle rate showed a linear increasing trend with increasing levels of the GPS-HCEO complex (0.05 < *P* (Linear) < 0.1). Additionally, the abdominal fat rate in Group A was significantly lower than in Group C and the Groups M1, M2, and M3 (*P* < 0.05), while the abdominal fat rates in Groups M1, M2, and M3 were comparable to Group C(*P* > 0.05).

**Table 3:** Effect of GPS and HCEO complex on slaughter performance of broilers.

Item	Treatment group					***P-value***		
	C	A	M1	M2	M3	ANOVA	Linear	Quadratic
SR (%)	83.82 ± 1.64	83.27 ± 0.68	83.25 ± 0.70	83.11 ± 1.64	84.29 ± 0.43	0.694	0.699	0.253
HEW (%)	77.30 ± 2.04	76.65 ± 0.70	76.62 ± 0.47	75.51 ± 1.21	76.91 ± 0.84	0.477	0.508	0.196
EW (%)	64.22 ± 1.74	63.30 ± 0.34	63.58 ± 0.58	62.24 ± 1.32	63.94 ± 0.32	0.309	0.480	0.114
LMR (%)	25.96 ± 1.34	23.79 ± 2.34	26.68 ± 1.48	27.53 ± 2.91	29.25 ± 4.96	0.295	0.216	0.859
PMR (%)	31.45 ± 0.58	33.07 ± 1.21	31.72 ± 1.48	30.80 ± 1.76	33.04 ± 0.25	0.152	0.244	0.189
LMP (%)	57.41 ± 1.57	56.86 ± 2.53	58.40 ± 1.75	58.33 ± 2.96	62.29 ± 4.76	0.262	0.099	0.437
WR (%)	8.69 ± 0.34	9.48 ± 0.97	9.17 ± 0.47	9.18 ± 0.56	9.32 ± 0.06	0.303	0.110	0.482
AFR (%)	2.44 ± 0.39^a^	1.11 ± 0.08^b^	2.00 ± 0.48^a^	2.23 ± 0.21^a^	2.05 ± 0.27^a^	0.005	0.340	0.565

Data are presented as least squares means based on 3 replicate observations per treatment, with each observation representing the average value of the corresponding measurements from one male and one female bird within each replicate. SR, slaughter rate; HEW, half-eviscerated weight; EW, eviscerated weight; PMR, pectoral muscle rate; LMR, leg muscle rate; AFR, abdominal fat rate; LMP, Lean meat percentage; WR, Wing rate; AFR, Abdominal fat rate. ^1)^C, a blank control (basal diets); A, an antibiotic group (basal diets +50 g/T chlortetracycline + 50 g/T salinomycin); M1, basal diets+ GPS and HCEO complex via drinking water at 0.1 ml/L; M2, basal diets+ GPS and HCEO complex via drinking water at 0.3 ml/L; M3, basal diets+ GPS and HCEO complex via drinking water at 0.6 ml/L. ^2)^The total *p*-value was obtained from the ANOVA analysis conducted on the 5 groups. The *p*-values for the linear and quadratic terms were derived from the ANOVA analysis performed on the control group and the 3 dose groups. ^a, b^Means within a variable with no common superscript differ significantly (*p* < 0.05).

### Serum biochemical indices

Compared to Group C (Table 4), Group A and Groups M1, M2, and M3 showed no significant effects on serum ALT, AST, TC, CREA, UREA, Glu-G, TP, ALB, or Glo levels (*P*> 0.05). However, serum TG levels in these four groups were significantly higher than in Group C (*P* < 0.05).

**Table 4:** Effect of GPS and HCEO complex on serum biochemical indices of broilers.

Item	Treatment group					***P-value***		
	C	A	M1	M2	M3	ANOVA	Linear	Quadratic
ALT (U/L)	2.60 ± 0.10	3.00 ± 0.79	2.13 ± 0.15	2.03 ± 1.15	2.10 ± 0.90	0.486	0.424	0.548
AST (U/L)	318.33 ± 52.81	259.93 ± 17.99	315.53 ± 33.37	320.90 ± 39.94	356.07 ± 61.44	0.194	0.368	0.514
TC (mmol/L)	3.82 ± 0.66	3.15 ± 0.57	2.92 ± 0.54	2.80 ± 0.28	3.32 ± 0.14	0.156	0.208	0.026
CREA (μmol/L)	11.00 ± 2.96	17.27 ± 15.63	13.97 ± 3.33	18.43 ± 7.04	11.90 ± 3.14	0.743	0.550	0.102
UREA (mmol/L)	0.42 ± 0.03	0.44 ± 0.09	0.62 ± 0.09	0.56 ± 0.16	0.70 ± 0.16	0.634	0.040	0.652
Glu-G (mmol/L)	12.31 ± 1.55	11.03 ± 1.53	10.31 ± 0.23	12.14 ± 1.37	10.59 ± 0.40	0.205	0.258	0.723
TG (mmol/L)	0.26 ± 0.01^c^	0.36 ± 0.04^b^	0.45 ± 0.04^a^	0.43 ± 0.06^ab^	0.47 ± 0.06^a^	0.001	0.001	0.022
TP (g/L)	27.87 ± 2.15	27.97 ± 2.93	28.70 ± 3.20	26.30 ± 2.34	28.73 ± 1.93	0.772	0.976	0.588
ALB (g/L)	10.70 ± 0.96	10.67 ± 0.85	11.13 ± 0.85	10.00 ± 1.13	10.70 ± 0.85	0.693	0.658	0.815
Glo (g/L)	17.17 ± 1.47	17.30 ± 2.10	17.57 ± 2.36	16.30 ± 1.21	18.03 ± 1.18	0.801	0.759	0.498

Data are presented as least squares means based on 3 replicate observations per treatment, with each observation representing the average value of the corresponding measurements from one male and one female bird within each replicate. ^1)^C, a blank control (basal diets); A, an antibiotic group (basal diets +50 g/T chlortetracycline + 50 g/T salinomycin); M1, basal diets+ GPS and HCEO complex via drinking water at 0.1 ml/L; M2, basal diets+ GPS and HCEO complex via drinking water at 0.3 ml/L; M3, basal diets+ GPS and HCEO complex via drinking water at 0.6 ml/L. ^2)^The total *p*-value was obtained from the ANOVA analysis conducted on the 5 groups. The *p*-values for the linear and quadratic terms were derived from the ANOVA analysis performed on the control group and the 3 dose groups. ^a−*c*^Means within a variable with no common superscript differ significantly (*p* < 0.05).

### Immune indices and inflammatory factors

Compared to Group C (Table 5), there were no significant differences in serum immune indices (IgM, IgG, IgA) or inflammatory factors (IL-1, IL-10) in the other four experimental groups (*P* > 0.05).

**Table 5:** Effect of GPS and HCEO complex on serum immune indices and inflammatory factors of broilers.

Item	Treatment group					***P-value***		
	C	A	M1	M2	M3	ANOVA	Linear	Quadratic
IgM (ug/ml)	22.33 ± 3.05	23.16 ± 0.45	24.17 ± 4.21	23.79 ± 0.37	25.53 ± 4.41	0.767	0.326	0.979
IgG (ug/ml)	256.24 ± 10.29	265.00 ± 10.29	272.88 ± 40.72	255.34 ± 13.36	271.88 ± 23.91	0.835	0.662	0.997
IgA (ug/ml)	28.55 ± 0.83	30.03 ± 2.81	27.03 ± 3.49	28.02 ± 0.20	27.51 ± 3.28	0.743	0.744	0.730
IL-1 (pg/ml)	49.49 ± 6.21	51.37 ± 0.68	61.66 ± 8.00	53.08 ± 3.72	61.97 ± 14.05	0.236	0.243	0.757
IL-10 (pg/ml)	158.48 ± 17.56	178.78 ± 14.55	149.61 ± 13.67	141.61 ± 8.49	133.63 ± 35.72	0.148	0.175	0.972

Data are presented as least squares means based on 3 replicate observations per treatment, with each observation representing the average value of the corresponding measurements from one male and one female bird within each replicate. IgM, Immunoglobulin M; IgG, Immunoglobulin G; IgA, Immunoglobulin A. ^1)^C, a blank control (basal diets); A, an antibiotic group (basal diets +50 g/T chlortetracycline + 50 g/T salinomycin); M1, basal diets+ GPS and HCEO complex *via* drinking water at 0.1 ml/L; M2, basal diets+ GPS and HCEO complex *via* drinking water at 0.3 ml/L; M3, basal diets+ GPS and HCEO complex via drinking water at 0.6 ml/L. ^2)^The total *p*-value was obtained from the ANOVA analysis conducted on the 5 groups. The *p*-values for the linear and quadratic terms were derived from the ANOVA analysis performed on the control group and the 3 dose groups.

### Intestinal morphology

As shown in Table 6, in the duodenum, compared with Group C, no significant differences were observed in VH or CD among the four treatment groups (*P* > 0.05), whereas the V/C ratios in Groups A and M2 were significantly lower than that in Group C (*P* < 0.05).

**Table 6:** Effect of GPS and HCEO complex on intestinal morphology of broilers.

Item		Treatment group					***P-value***		
		C	A	M1	M2	M3	ANOVA	Linear	Quadratic
Duodenum	VH (μm)	1,583.37 ± 30.88^ab^	1,213.11 ± 205.87^b^	1,473.09 ± 233.63^b^	1,413.73 ± 78.31^b^	1,840.16 ± 285.78^a^	0.028	0.184	0.040
	CD (μm)	260.02 ± 52.84	337.13 ± 50.93	286.02 ± 2.28	349.87 ± 38.53	293.03 ± 67.68	0.201	0.217	0.166
	V/C	6.31 ± 1.34^a^	3.71 ± 0.88^b^	5.21 ± 0.79^ab^	4.07 ± 0.33^b^	6.39 ± 0.43^a^	0.007	0.681	0.007
Jejunum	VH (μm)	1,182.34 ± 74.28^b^	1,152.91 ± 147.42^b^	1,258.52 ± 175.92^b^	1,240.82 ± 103.89^b^	1,647.00 ± 308.37^a^	0.043	0.022	0.168
	CD (μm)	307.87 ± 25.56	379.94 ± 55.44	321.65 ± 52.50	317.85 ± 76.82	246.50 ± 56.66	0.146	0.230	0.224
	V/C	3.87 ± 0.44^b^	3.14 ± 0.89^b^	3.94 ± 0.31^b^	4.11 ± 1.07^b^	7.03 ± 2.34^a^	0.043	0.036	0.131
Ileum	VH (μm)	874.18 ± 80.13	853.81 ± 147.38	898.34 ± 275.54	844.59 ± 83.16	1,046.52 ± 431.39	0.842	0.513	0.574
	CD (μm)	205.28 ± 92.53	139.16 ± 28.65	273.36 ± 91.53	151.59 ± 29.36	119.12 ± 32.53	0.070	0.030	0.141
	V/C	4.80 ± 1.83^b^	6.31 ± 1.35^ab^	3.56 ± 1.75^b^	5.82 ± 1.62^ab^	8.99 ± 2.46^a^	0.044	0.018	0.085

Data are presented as least squares means based on 3 replicate observations per treatment, with each observation representing the average value of the corresponding measurements from one male and one female bird within each replicate. VH, villus height; CD, crypt depth; V/C, villus height to crypt depth ratio; SEM, standard error of means. ^1)^C, a blank control (basal diets); A, an antibiotic group (basal diets +50 g/T chlortetracycline + 50 g/T salinomycin); M1, basal diets+ GPS and HCEO complex via drinking water at 0.1 ml/L; M2, basal diets+ GPS and HCEO complex via drinking water at 0.3 ml/L; M3, basal diets+ GPS and HCEO complex via drinking water at 0.6 ml/L. ^2)^The total *p*-value was obtained from the ANOVA analysis conducted on the 5 groups. The *p*-values for the linear and quadratic terms were derived from the ANOVA analysis performed on the control group and the 3 dose groups. ^a, b^Means within a variable with no common superscript differ significantly (*p* < 0.05).

In the jejunum, compared with Group C, Groups A, M1, and M2 showed no significant differences in VH, CD, or V/C (*P* > 0.05), whereas Group M3 showed significantly increased VH and V/C (*P* < 0.05).

In the ileum, compared with Group C, no significant differences were observed in VH or CD among the four treatment groups (*P* > 0.05). The V/C ratios in Groups M1, M2, and M3 were comparable to that in Group A (*P* > 0.05), whereas Group M3 showed a significantly higher V/C ratio than Group C (*P* < 0.05).

### Cecal microbiota

#### α-diversity

**Figure 1:**
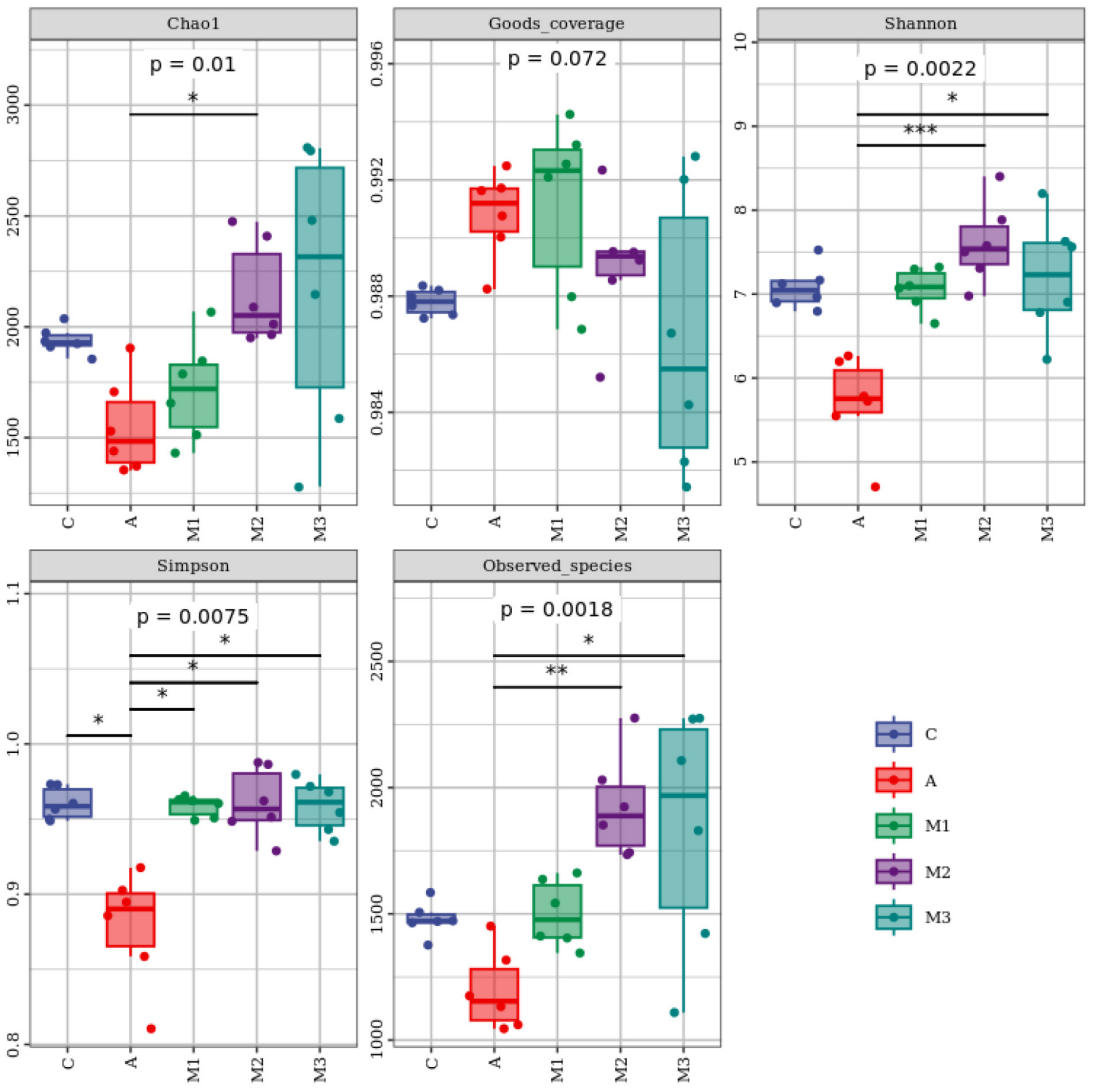
Effect of GPS and HCEO complex on α-diversity of cecal microbiota in broilers. ^1)^C, a blank control (basal diets); A, an antibiotic group (basal diets +50 g/T chlortetracycline + 50 g/T salinomycin); M1, basal diets+ GPS and HCEO complex via drinking water at 0.1 ml/L; M2, basal diets+ GPS and HCEO complex via drinking water at 0.3 ml/L; M3, basal diets+ GPS and HCEO complex via drinking water at 0.6 ml/L. *, **, ***: significant difference (*P* < 0.05), *P < 0.05, **P < 0.01, ***P < 0.001.

As shown in Figure 1, compared with Group C, the Simpson index was significantly decreased in Group A (*P* < 0.05). The Simpson index in Group M1 was significantly higher than that in Group A (*P* < 0.05). In Group M2, the Chao1, Shannon, Simpson, and Observed_species indices were significantly higher than those in Group A (*P* < 0.05). Similarly, the Shannon, Simpson, and Observed_species indices in Group M3 were also significantly higher than those in Group A (*P* < 0.05). However, compared with Group C, no significant differences in α-diversity indices were observed in Groups M1, M2, or M3 (*P* > 0.05).

### Microbiota composition

**Figure 2:**
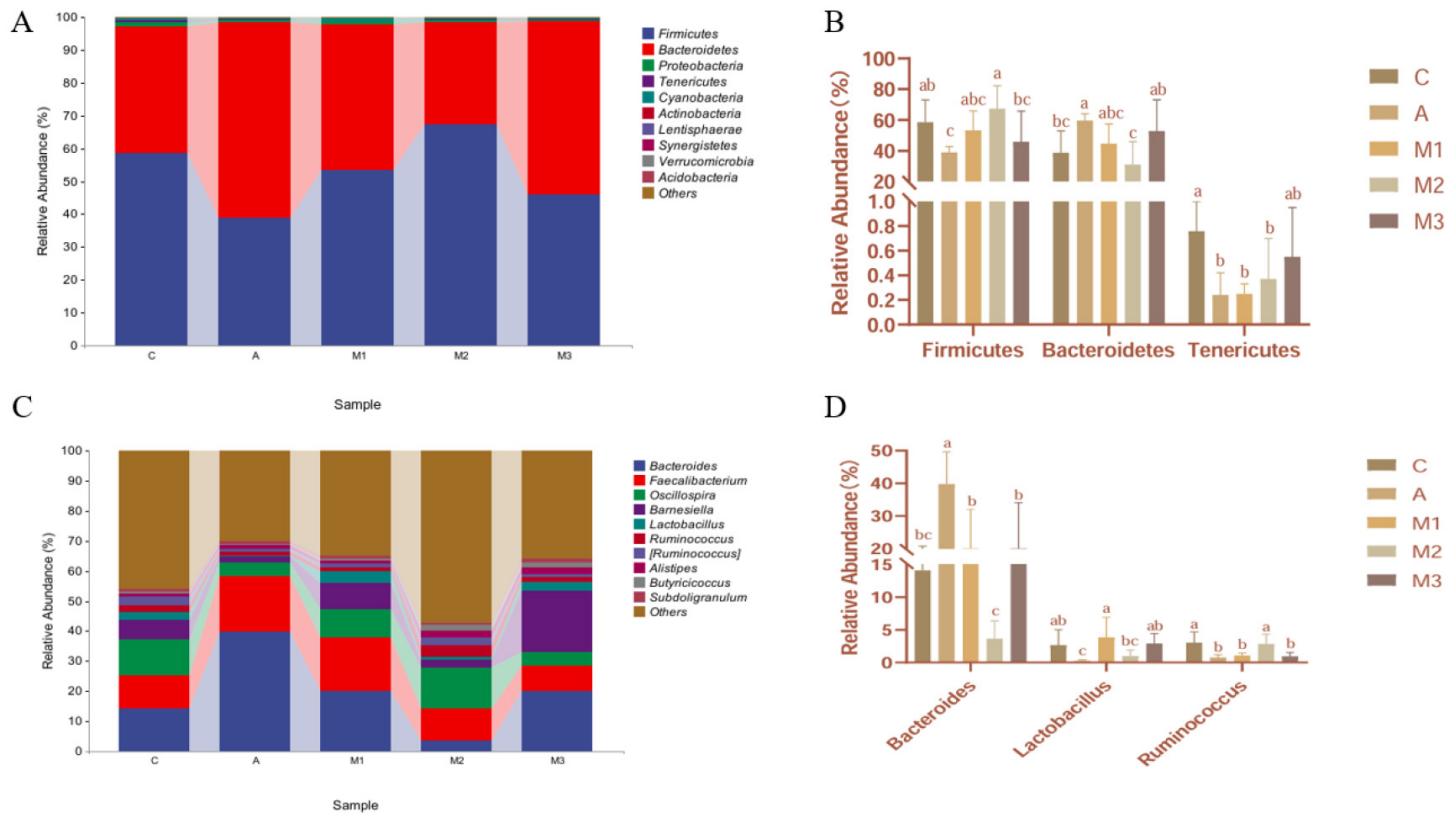
Effect of GPS and HCEO complex on cecal microbiota composition in broilers. ^1)^C, a blank control (basal diets); A, an antibiotic group (basal diets +50 g/T chlortetracycline + 50 g/T salinomycin); M1, basal diets+ GPS and HCEO complex via drinking water at 0.1 ml/L; M2, basal diets+ GPS and HCEO complex via drinking water at 0.3 ml/L; M3, basal diets+ GPS and HCEO complex via drinking water at 0.6 ml/L. ^a−*c*^Means within a variable with no common superscript differ significantly (*p* < 0.05).

At the phylum level (Top 10, Figures 2A, B), compared to Group C, Group A showed a significant decrease in the abundance of *Firmicutes* and *Tenericutes*, and a significant increase in *Bacteroidetes* (*P* < 0.05). Groups M1, M2, and M3 showed no significant difference in *Firmicutes* or *Tenericutes* abundance (*P* > 0.05). Group M2 had significantly higher *Firmicutes* abundance and significantly lower *Bacteroidete*s abundance than Group A (*P* < 0.05). Regarding *Tenericutes*, M1 and M2 were significantly lower than Group C (*P* < 0.05), but M3 was comparable to Group C.

At the genus level (Top 10, Figures 2C, D), compared to Group C, Group A showed a significant increase in *Bacteroides* and a significant decrease in *Lactobacillus* and *Ruminococcus* (*P* < 0.05). Groups M1, M2, and M3 showed no significant differences in B*acteroides* or *Lactobacillus* abundance (*P* > 0.05). For *Ruminococcus*, M1 and M3 were significantly lower than Group C (*P* < 0.05), but M2 was comparable to Group C.

### LEfSe analysis

**Figure 3:**
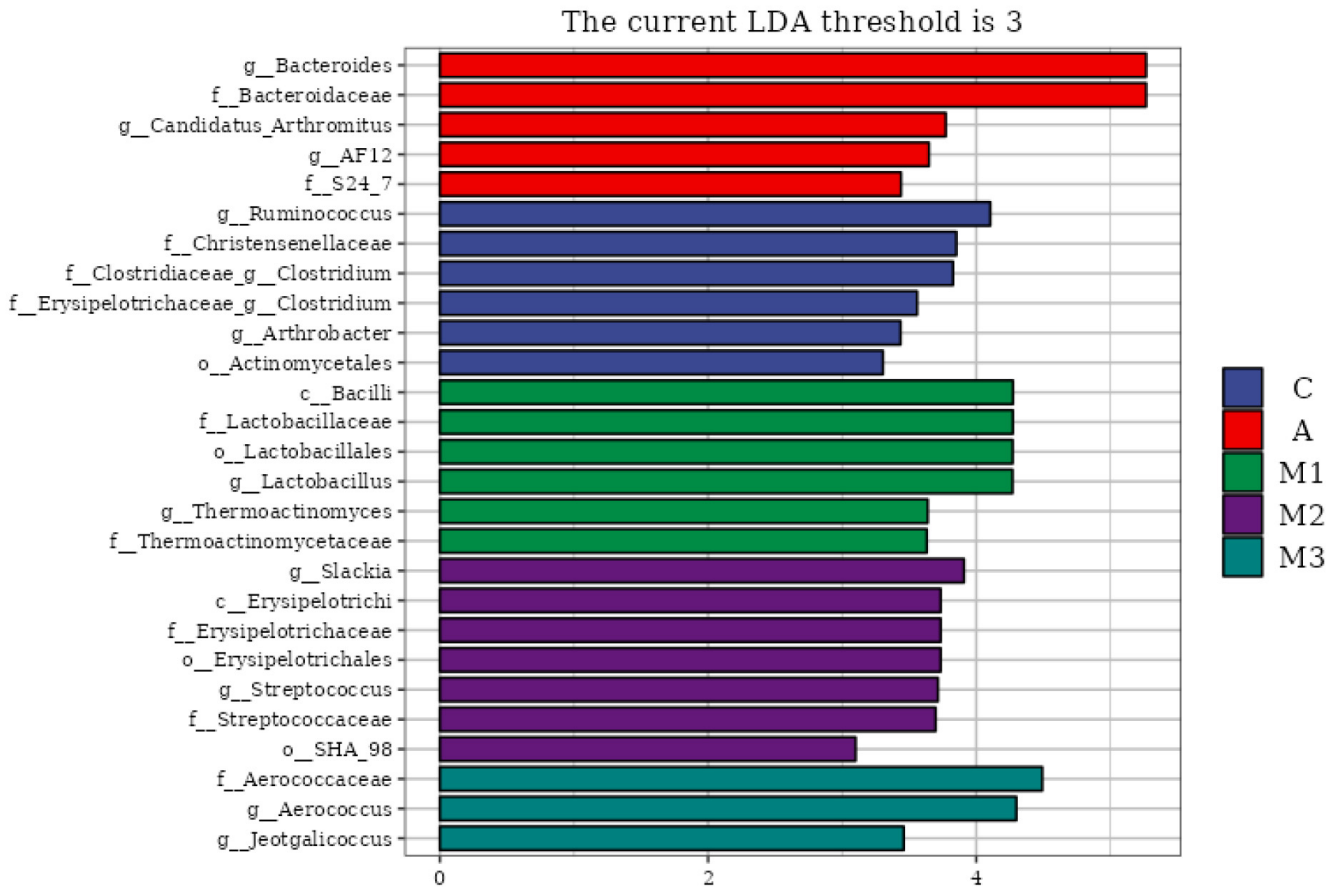
Linear discriminant analysis effect size (LEfSe) analysis integrated with linear discriminant analysis (LDA) revealed differentially abundant phylotypes in different groups. ^1)^C, a blank control (basal diets); A, an antibiotic group (basal diets +50 g/T chlortetracycline + 50 g/T salinomycin); M1, basal diets+ GPS and HCEO complex via drinking water at 0.1 ml/L; M2, basal diets+ GPS and HCEO complex via drinking water at 0.3 ml/L; M3, basal diets+ GPS and HCEO complex via drinking water at 0.6 ml/L.

As shown in Figure 3, differential bacterial enrichment was observed in all five experimental groups when the LDA score threshold was set at >3. At the genus level, the differentially enriched bacteria in Group C mainly included *Bacteroides, Candidatus_Arthromitus*, and *AF12*. In Group A, the enriched genera were mainly *Ruminococcus, Clostridium*, and *Arthrobacter*. In Group M1, the enriched genera were mainly *Lactobacillus* and *Thermoactinomyces*. The differentially enriched genera in Group M2 were *Slackia* and *Streptococcus*, whereas those in Group M3 were *Aerococcus* and *Jeotgalicoccus*.

## Discussion

Growth performance is a key indicator for evaluating the economic benefits of livestock breeding, with the FCR intuitively reflecting growth efficiency (12). Behboodi et al. reported that supplementation with 0.2–0.5 ml/L ImmunoMax, a plant extract-based product, in drinking water improved the growth performance of broilers (13). Liu (5) also confirmed that GPS improved FCR in broilers. Plant essential oils have shown similar effects. Adaszynska-Skwirzynska et al. found that supplementation with 0.4 ml/L plant essential oils in drinking water significantly improved feed conversion efficiency in broilers aged 22–42 days (14). Xie (8) observed in goats that houttuynia cordata extract reduced FCR by increasing crude protein and fiber digestibility. Consistent with these previous findings, the present study showed that dietary supplementation with a high dose of GPS-HCEO complex significantly reduced FCR during the early, late, and overall experimental periods. GPS, as a prebiotic, can promote the proliferation of short-chain fatty acid-producing bacteria in the intestine, thereby providing energy substrates such as propionate and butyrate for intestinal epithelial cell development (15). HCEO can inhibit the growth of harmful intestinal bacteria and reduce intestinal inflammation (16), creating a favorable microenvironment for intestinal epithelial development. Therefore, the improvement in FCR induced by the GPS-HCEO complex may be attributed to the synergistic effects of GPS and HCEO in promoting intestinal epithelial development and enhancing nutrient absorption.

Slaughter performance is a crucial metric for production efficiency (17). In the present study, compared with the control group, leg muscle yield tended to increase linearly with increasing dietary levels of the GPS-HCEO complex, and Group M3 showed a 4.88% increase compared with the control group. This aligns with Zheng (18) found that Yupingfeng polysaccharide increased leg muscle yield in Qingyuan partridge chickens by 16.8%. The increase in leg muscle yield by the GPS-HCEO complex may be attributed to GPS reducing skeletal muscle fiber apoptosis (19) or essential oil components promoting muscle hypertrophy by increasing fiber area and diameter (20). Additionally, while the abdominal fat rate of healthy Arbor Acres broilers is generally maintained above 1.55% (21), the antibiotic group in this study showed a rate of only 1.11%, significantly lower than the control and GPS-HCEO groups. This may be because long-term antibiotic use inhibits the differentiation of preadipocytes into adipocytes, resulting in excessively low abdominal fat deposition (22).

Serum biochemical indices directly reflect animal metabolism and health status (23), with TG content indicating lipid metabolism. In this study, both the antibiotic group and the GPS-HCEO groups significantly increased serum TG levels (*P* < 0.05), suggesting both participate in lipid metabolism regulation. Lei (24) reported that antibiotics can interfere with lipid metabolism through multiple mechanisms and generally lead to elevated serum TG levels. Similarly, Bormon et al. (25) found that antibiotics increased serum TG content in broilers, which is consistent with the results of the present study. This phenomenon may be attributed to antibiotic-induced disruption of the gut microbiota, leading to an increase in bacterial populations associated with fat absorption (26). Kwak et al. reported that GPS reduced the elevated serum TG level in an endogenous hyperlipidemia model by increasing the activity of lipoprotein lipase (LPL) in serum (27). Kang et al. found that Houttuynia cordata extract attenuated the high-fat diet-induced increase in serum TG levels by enhancing AMPK phosphorylation (28). In addition, previous studies have shown that plant polysaccharides and essential oils can improve intestinal morphology and promote fat absorption (29, 30). The regulatory effects of the GPS-HCEO complex on serum TG levels appear to be inconsistent with some previous findings, which may be related to differences in experimental models. In the present study, the dietary fat level of broilers was relatively low. However, since both the GPS-HCEO complex and antibiotics increased serum TG levels in broilers, further studies are needed to examine the expression of lipid metabolism-related genes and liver morphology to determine whether the GPS-HCEO complex improves or disrupts lipid metabolism.

Cytokines are crucial for evaluating immune function and intestinal barrier health (31). Liou et al. reported that ginseng extract dose-dependently increased serum IgG, IL-2, and IFN-γ levels (32). In addition, Liu (5) found that supplementation with 200 g/T GPS increased serum IgG levels and decreased the levels of pro-inflammatory cytokines, including TNF-α and IL-6, in broilers. Unlike these previous findings, the present study showed that dietary supplementation with the GPS-HCEO complex did not significantly affect serum IgG, IgM, IgA, IL-1, or IL-10 levels in broilers. This may be because the supplementation level used in this study was insufficient to exert a marked immunomodulatory effect. Yan (10) also found that Houttuynia cordata extract did not affect serum IgG levels or lymphocyte proportions in pigs, which is consistent with the present results and suggests that the GPS-HCEO complex may have limited stimulatory effects on systemic humoral immunity. Furthermore, the broilers used in the present study were healthy and were not subjected to pathogen challenge. Under normal physiological conditions, the immune system is tightly regulated by homeostatic mechanisms, and serum IgG, IgM, IgA, IL-1, and IL-10 levels may remain relatively stable or low. Therefore, the immunomodulatory effects of dietary immune regulators may be limited in healthy animals. Future studies should consider increasing the supplementation level of the GPS-HCEO complex and conducting bacterial challenge experiments to further investigate its effects on immune function in broilers.

Intestinal morphology is vital for gut health. VH reflects intestinal absorptive capacity, CD reflects stem cell activity, and the V/C ratio is positively correlated with growth rate (33). In the present study, antibiotics reduced the duodenal V/C ratio in broilers, but had limited effects on the jejunum and ileum. Zhang et al. reported that long-term antibiotic use can impair the nutrient absorption structure of the duodenum (34), which is consistent with the findings of the present study. In addition, antibiotics mainly affect host health by disrupting the gut microbiota (35). The duodenum is an important site for bacterial colonization in the small intestine, with higher bacterial abundance and diversity than the jejunum and ileum (36). Therefore, the inconsistent effects of antibiotics on intestinal morphology across different intestinal segments may be related to differences in microbial abundance and diversity among intestinal segments. In contrast, Selim (37) found that 300 g/T plant essential oil improved VH and CD, and Liu (5) observed that GPS increased intestinal V/C. Consistent with these studies, the present study confirmed that dietary supplementation with a high dose of the GPS-HCEO complex significantly improved jejunal VH and V/C ratio, as well as ileal V/C ratio. These results indicate that high-dose GPS-HCEO complex administration can effectively improve intestinal morphology, promoting the absorption of amino acids, carbohydrates, and fats, which is likely a key reason for the observed improvements in leg muscle rate and serum TG levels. It has been reported that the absorption of polysaccharides differs among intestinal segments, with plant polysaccharide absorption mainly occurring in the ileum (38). Ocelová et al. also found that the concentration of plant essential oils in the jejunum of broilers was higher than that in the duodenum (39). This may explain why the GPS-HCEO complex mainly affected intestinal morphology in the jejunum and ileum, but had a relatively limited effect on duodenal morphology. Further studies are needed to verify the absorption and distribution characteristics of the GPS-HCEO complex in different intestinal segments.

Physiology and metabolism are regulated by the microbiome. McDonnell (40) found via meta-analysis that antibiotic exposure reduces microbiota diversity and richness. Our results show that antibiotics significantly reduced cecal Simpson indices, confirming the disruptive effect of antibiotics on the microbiota. Bacteria of the phyla *Firmicutes* and *Tenericutes* are integral to a healthy gut; reduced *Firmicutes* abundance is often associated with infection and inflammatory bowel disease (41), while decreased *Tenericutes* correlates with barrier dysfunction (42). *Bacteroidetes* are dual-natured, some aid polysaccharide digestion, while others produce toxins that damage the epithelial barrier (43). Our results show that antibiotics significantly reduced *Firmicutes* and *Tenericutes* and increased *Bacteroidetes*, indicating that long-term antibiotic use facilitates the proliferation of potentially harmful bacteria (44). In contrast, Group M3 did not significantly alter the top 10 phyla compared to Group C, suggesting the complex has a milder impact on the microbiota. The LEfSe analysis showed similar results. Antibiotic treatment enriched the potentially harmful genera *Clostridium* and *Arthrobacter* in the intestine. The enrichment of *Clostridium* is directly associated with the occurrence of necrotic enteritis in broilers (45), whereas Arthrobacter is an environmental microorganism closely related to milk spoilage (46). In Group M3, the differentially enriched genera were *Aerococcus* and *Jeotgalicoccus*. *Aerococcus* can ferment carbohydrates and produce lactic acid (47), while *Jeotgalicoccus* has been associated with short-chain fatty acid production (48). These findings suggest that high-dose supplementation with the GPS-HCEO complex may promote the enrichment of beneficial intestinal bacteria to some extent, which is consistent with the findings of Zong et al. (4).

### Study restrictions

This study systematically investigated the effects of antibiotics and the GPS-HCEO complex on growth performance, slaughter performance, serum biochemical indices, immune function, intestinal morphology, and cecal microbiota in broilers. Although linear and quadratic analyses were performed using the control group and the GPS-HCEO complex groups, and the results supported the application potential of the GPS-HCEO complex in broilers, differences in the administration methods between antibiotics and the GPS-HCEO complex may still affect the direct comparison of their effects. In addition, the present experiment included only three replicates, and the sample size met only the minimum requirement. Therefore, further studies with larger sample sizes are needed to validate these findings. Therefore, further optimization studies using different broiler breeds are warranted.

## Conclusion

In summary, while long-term antibiotic supplementation in feed reduced the FCR in Arbor Acres broilers, it also excessively inhibited abdominal fat deposition, reduced V/C, and caused a decline in the abundance of beneficial bacteria (*Firmicutes, Tenericutes*). Administration of 0.6 ml/L of the Panax ginseng polysaccharide and Houttuynia cordata essential oil complex achieved an FCR comparable to the antibiotic group, promoted the development of the jejunum, and ileum, and improved cecal microbiota structure to a certain extent.

## Data Availability

The data that support the findings of this study are available from Hunan Agricultural University, but restrictions apply to the availability of these data, which were used under license for the current study and so are not publicly available. The data are, however, available from the authors upon reasonable request and with the permission of Hunan Agricultural University. Requests to access the datasets should be directed to lzk7035@sina.com.

## References

[1] Guo M Shao S Wang D Zhao D Wang M. Recent progress in polysaccharides from Panax ginseng C. A Meyer. Food Funct. (2021) 12:494–518. doi: 10.1039/D0FO01896A 33331377

[2] Islam MM. Bacterial resistance to antibiotics: access, excess, and awareness in Bangladesh. Expert Rev Anti Infect Ther. (2021) 19:973–81. doi: 10.1080/14787210.2021.1865804 33353447

[3] Muaz K Riaz M Akhtar S Park S Ismail A. Antibiotic residues in chicken meat: global prevalence, threats, and decontamination strategies: a review. J Food Prot. (2018) 81:619–27. doi: 10.4315/0362-028X.JFP-17-086 29537307

[4] Zong G Deng R Pan Y Liu M Zhu H Tao R . Ginseng polysaccharides ameliorate colorectal tumorigenesis through Lachnospiraceae-mediated immune modulation. Int J Biol Macromol. (2025) 307:142015. doi: 10.1016/j.ijbiomac.2025.142015 40081698

[5] Liu J Wang H Luo J Chen T Xi Q Sun J . Synergism of fermented feed and ginseng polysaccharide on growth performance, intestinal development, and immunity of Xuefeng black-bone chickens. BMC Vet Res. (2024) 20:13. doi: 10.1186/s12917-023-03859-y 38184589 PMC10770880

[6] Yang CM Han QJ Wang KL Xu YL Lan JH Cao GT. Astragalus and ginseng polysaccharides improve developmental, intestinal morphological, and immune functional characters of weaned piglets. Front Physiol. (2019) 10:418. doi: 10.3389/fphys.2019.00418 31031640 PMC6473041

[7] Wang S Li L Chen Y Liu Q Zhou S Li N . Houttuynia cordata thunb. alleviates inflammatory bowel disease by modulating intestinal microenvironment: a research review. Front Immunol. (2023) 14:1306375. doi: 10.3389/fimmu.2023.1306375 38077358 PMC10702737

[8] Xie L Jian Z Tang M Li C Huang Z Wang F . Effects of summer supplementation of *Houttuynia cordata* extract on growth performance, anti-inflammatory properties, and rumen fermentation in Guizhou black goats. Front Vet Sci. (2025) 12:1627331. doi: 10.3389/fvets.2025.1627331 40678498 PMC12268708

[9] Jung BG Ko JH Lee BJ. Dietary supplementation with a probiotic fermented four-herb combination enhances immune activity in broiler chicks and increases survivability against Salmonella Gallinarum in experimentally infected broiler chicks. J Vet Med Sci. (2010) 72:1565–73. doi: 10.1292/jvms.10-0152 20675965

[10] Yan L Zhang ZF Park JC Kim IH. Evaluation of houttuynia cordata and taraxacum officinale on growth performance, nutrient digestibility, blood characteristics, and fecal microbial shedding in diet for weaning pigs. Asian-Australas J Anim Sci. (2012) 25:1439–44. doi: 10.5713/ajas.2012.12215 25049500 PMC4093006

[11] Wang F Yin Y Yang M Chen J Fu C Huang K. Effects of combined supplementation of *macleaya cordata* extract and *benzoic acid* on the growth performance, immune responses, antioxidant capacity, intestinal morphology, and microbial composition in weaned piglets. Front Vet Sci. (2021) 8:708597. doi: 10.3389/fvets.2021.708597 34490398 PMC8416536

[12] Zhao Y Jin C Xuan Y Zhou P Fang Z Che L . Effect of maternal or post-weaning methyl donor supplementation on growth performance, carcass traits, and meat quality of pig offspring. J Sci Food Agric. (2019) 99:2096–107. doi: 10.1002/jsfa.9402 30298675

[13] Behboodi HR Hosseini D Salarieh A Gholampour M Panahi M Alemi M . Impact of drinking water supplementation of a blend of peppermint, coneflower (Echinacea purpurea), thyme, propolis, and prebiotic on performance, serum constituents, and immunocompetence of broiler chickens. Trop Anim Health Prod. (2022) 54:289. doi: 10.1007/s11250-022-03274-9 36088511

[14] Adaszynska-Skwirzynska M Szczerbinska D. The effect of lavender (Lavandula angustifolia) essential oil as a drinking water supplement on the production performance, blood biochemical parameters, and ileal microflora in broiler chickens. Poult Sci. (2019) 98:358–65. doi: 10.3382/ps/pey385 30165505

[15] Li C Zhou S Chen S Xu Z Xue Q Wang H . Gut microbiota-mediated immunomodulation underlies the anti-tumor effects of a novel ginseng polysaccharide. Carbohydr Polym. (2025) 370:124458. doi: 10.1016/j.carbpol.2025.124458 41116580

[16] Wu Z Deng X Hu Q Xiao X Jiang J Ma X . *Houttuynia cordata* Thunb: an ethnopharmacological review. Front Pharmacol. (2021) 12:714694. doi: 10.3389/fphar.2021.714694 34539401 PMC8440972

[17] Yan X Xu Y Zhen Z Li J Zheng H Li S . Slaughter performance of the main goose breeds raised commercially in China and nutritional value of the meats of the goose breeds: a systematic review. J Sci Food Agric. (2023) 103:3748–60. doi: 10.1002/jsfa.12244 36178068

[18] Zheng W Chen S Guan Y Wu B. Effects of Yupingfeng polysaccharide in diet on slaughtering performance and meat flavor of Qingyuan partridge chicken. Food Chem. (2025) 471:142814. doi: 10.1016/j.foodchem.2025.142814 39798377

[19] Trushina EN Mustafina OK Aksenov IV Krasutsky AG Tutelyan VA Nikityuk DB. Protective action of ginseng root extract on myofibril apoptosis and immune response in rats after exhausting physical exercise. Part I Effect of ginseng root extract on myofibril apoptosis in rats' gastrocnemius muscle. Vopr Pitan. (2025) 94:110–7. doi: 10.33029/0042-8833-2025-94-1-111-117 40214687

[20] Terruzzi I Vacante F Senesi P Montesano A Codella R Luzi L. Effect of hazelnut oil on muscle cell signalling and differentiation. J Oleo Sci. (2018) 67:1315–26. doi: 10.5650/jos.ess18086 30210078

[21] Sarsenbek A Wang T Zhao JK Jiang W. Comparison of carcass yields and meat quality between Baicheng-You chickens and Arbor Acres broilers. Poult Sci. (2013) 92:2776–82. doi: 10.3382/ps.2012-02841 24046427

[22] Lei YY. Research on the interference effects and mechanisms of four typical antibiotics on body lipid metabolism. Changchun: China Jilin University (2022).

[23] Ning Z Wang H Qin R Wei W Aireti N Guo S . Effects of Lactobacillus plantarum-fermented feed and postbiotics on the growth performance, digestibility, serum biochemistry, and caecal microbiota of chickens. Poult Sci. (2025) 105:106275. doi: 10.1016/j.psj.2025.106275 41442915 PMC12799937

[24] Lei Y Li F Mortimer M Li Z Peng BX Li M . Antibiotics disrupt lipid metabolism in zebrafish (Danio rerio) larvae and 3T3-L1 preadipocytes. Sci Total Environ. (2023) 858:159755. doi: 10.1016/j.scitotenv.2022.159755 36349636

[25] Bormon CC Akib G Rifat A Hossain M Uddin N Hossain FMA . Effects of oyster mushroom (Pleurotus ostreatus) stem residue supplementation on growth performance, meat quality and health status of broilers. Poult Sci. (2024) 103:104054. doi: 10.1016/j.psj.2024.104054 39067124 PMC11337655

[26] Li HY Huang SY Zhou DD Xiong RG Luo M Saimaiti A . Theabrownin inhibits obesity and non-alcoholic fatty liver disease in mice via serotonin-related signaling pathways and gut-liver axis. J Adv Res. (2023) 52:59–72. doi: 10.1016/j.jare.2023.01.008 36639024 PMC10555776

[27] Kwak YS Kyung JS Kim JS Cho JY Rhee MH. Anti-hyperlipidemic effects of red ginseng acidic polysaccharide from Korean red ginseng. Biol Pharm Bull. (2010) 33:468–72. doi: 10.1248/bpb.33.468 20190411

[28] Kang H Koppula S. Houttuynia cordata alleviates high-fat diet-induced non-alcoholic fatty liver in experimental rats. Pharm Biol. (2015) 53:414–22. doi: 10.3109/13880209.2014.923002 25272018

[29] Manafu Z Du R Kudereti T Abulikemu G Lakho SA Xue L . Structure characterization and intestinal immune promotion effect of polysaccharide purified from Alhagi camelorum Fisch. Int J Biol Macromol. (2024) 269:132077. doi: 10.1016/j.ijbiomac.2024.132077 38723832

[30] Liu Z Mu Y Xing T Zhao L Li J Zhou J . Coated oregano essential oil and cinnamaldehyde compounds supplementation improves growth performance, enhances immune responses, and inhibits cecal Escherichia coli proliferation of broilers. J Anim Sci. (2024) 102:skae324. doi: 10.1093/jas/skae324 39434684 PMC11544625

[31] Wang Y Zhang J Wang X Wang R Zhang H Zhang R . The inflammatory immunity and gut microbiota are associated with fear response differences in laying hens. Poult Sci. (2024) 103:103816. doi: 10.1016/j.psj.2024.103816 38718537 PMC11097073

[32] Liou CJ Li ML Tseng J. Intraperitoneal injection of ginseng extract enhances both immunoglobulin and cytokine production in mice. Am J Chin Med. (2004) 32:75–88. doi: 10.1142/S0192415X04001771 15154287

[33] Liang J Zhong Z Wang A Yin Y Zheng K Zhou X. Lemongrass (*Cymbopogon citratus*) supplementation improves growth performance, intestinal function and inflammation status in weaned piglets. Anim Nutr. (2025) 24:61–73. doi: 10.1016/j.aninu.2025.09.007 41584688 PMC12828569

[34] Zhang Y Liu Y Jiao S Wang Y Sa R Zhao F . Short-term supplementation with uncoated and encapsulated Enterococcus faecium affected growth performance, gut microbiome and intestinal barrier integrity in broiler chickens. Poult Sci. (2024) 103:103808. doi: 10.1016/j.psj.2024.103808 38761463 PMC11133978

[35] Wu F Yu XA Angeles-Albores D Erdman SE Alm EJ. Sulfated dietary fiber protects gut microbiota from antibiotics. Microbiome. (2025) 13:183. doi: 10.1186/s40168-025-02176-w 40770727 PMC12329940

[36] Liao X Shao Y Sun G Yang Y Zhang L Guo Y . The relationship among gut microbiota, short-chain fatty acids, and intestinal morphology of growing and healthy broilers. Poult Sci. (2020) 99:5883–95. doi: 10.1016/j.psj.2020.08.033 33142506 PMC7647869

[37] Selim S Abdel-Megeid NS Alhotan RA Ebrahim A Hussein E. Nutraceuticals vs. antibiotic growth promoters: differential impacts on performance, meat quality, blood lipids, cecal microbiota, and organ histomorphology of broiler chicken. Poult Sci. (2024) 103:103971. doi: 10.1016/j.psj.2024.103971 38941788 PMC11260365

[38] Zheng Z Pan X Luo L Zhang Q Huang X Liu Y . Advances in oral absorption of polysaccharides: Mechanism, affecting factors, and improvement strategies. Carbohydr Polym. (2022) 282:119110. doi: 10.1016/j.carbpol.2022.119110 35123759

[39] Ocelová V Chizzola R Battelli G Pisarcikova J Faix S Gai F . Thymol in the intestinal tract of broiler chickens after sustained administration of thyme essential oil in feed. J Anim Physiol Anim Nutr. (2019) 103:204–9. doi: 10.1111/jpn.12995 30264416

[40] McDonnell L Gilkes A Ashworth M Rowland V Harries TH Armstrong D . Association between antibiotics and gut microbiome dysbiosis in children: systematic review and meta-analysis. Gut Microbes. (2021) 13:1–18. doi: 10.1080/19490976.2020.1870402 PMC7928022 33651651

[41] Akiyama S Nishijima S Kojima Y Kimura M Ohsugi M Ueki K . Multi-biome analysis identifies distinct gut microbial signatures and their crosstalk in ulcerative colitis and Crohn's disease. Nat Commun. (2024) 15:10291. doi: 10.1038/s41467-024-54797-8 39604394 PMC11603027

[42] Huang C Chen J Wang J Zhou H Lu Y Lou L . Dysbiosis of intestinal microbiota and decreased antimicrobial peptide level in paneth cells during hypertriglyceridemia-related acute necrotizing pancreatitis in rats. Front Microbiol. (2017) 8:776. doi: 10.3389/fmicb.2017.00776 28522995 PMC5415626

[43] Ge Y Huang K Xie W Xu C Yao Q Liu Y. Effects of *Rhodotorula mucilaginosa* on the Immune Function and Gut Microbiota of Mice. Front Fungal Biol. (2021) 2:705696. doi: 10.3389/ffunb.2021.705696 37744147 PMC10512290

[44] Buthasane P Roytrakul S Phaonakrop N Tunsagool P Buthasane W Am-In N . Metaproteomic analysis of gut resistome in the cecal microbiota of fattening pigs raised without antibiotics. Microbiol Spectr. (2023) 11:e0222323. doi: 10.1128/spectrum.02223-23 37439677 PMC10433946

[45] Gautam H Ayalew LE Shaik NA Subhasinghe I Popowich S Chow-Lockerbie B . Exploring the predictive power of jejunal microbiome composition in clinical and subclinical necrotic enteritis caused by Clostridium perfringens: insights from a broiler chicken model. J Transl Med. (2024) 22:80. doi: 10.1186/s12967-023-04728-w 38243294 PMC10799374

[46] Sutthiwong N Lekavat S Dufossé L. Involvement of versatile bacteria belonging to the genus *arthrobacter* in milk and dairy products. Foods. (2023) 12:1270. doi: 10.3390/foods12061270 36981196 PMC10048301

[47] Akpoghelie PO Edo GI Mafe AN Isoje EF Igbuku UA Ali ABM . Food, health, and environmental impact of lactic acid bacteria: the superbacteria for posterity. Probiotics Antimicrob Proteins. (2025) 17:2819–55. doi: 10.1007/s12602-025-10546-x 40289239

[48] Pei L Liu W Liu L Wang X Jiang L Chen Z . Morel (Morchella spp) intake alters gut microbial community and short-chain fatty acid profiles in mice. Front Nutr. (2023) 10:1237237. doi: 10.3389/fnut.2023.1237237 37810928 PMC10556497

